# Examining database persistence of ISO/EN 13606 standardized electronic health record extracts: relational vs. NoSQL approaches

**DOI:** 10.1186/s12911-017-0515-4

**Published:** 2017-08-18

**Authors:** Ricardo Sánchez-de-Madariaga, Adolfo Muñoz, Raimundo Lozano-Rubí, Pablo Serrano-Balazote, Antonio L. Castro, Oscar Moreno, Mario Pascual

**Affiliations:** 1 Telemedicine and Information Society Department, Health Institute “Carlos III” (ISCIII), c/Sinesio Delgado, 4 –, 28029 Madrid, Spain; 2 Medical Informatics, Hospital Clínic, Unit of Medical Informatics, University of Barcelona, Barcelona, Spain; 3 Department of Computer Science, Autonomous Univerity of Barcelona, Barcelona, Spain; 4 Doce de Octubre University Hospital, Madrid, Spain

**Keywords:** Relational database, NoSQL database, Normalized medical information, ISO/EN 13606 standard, Electronic health record extract, Algorithmic complexity, Primary use, Clinical practice, Secondary research use, Document-based task

## Abstract

**Background:**

The objective of this research is to compare the relational and non-relational (NoSQL) database systems approaches in order to store, recover, query and persist standardized medical information in the form of ISO/EN 13606 normalized Electronic Health Record XML extracts, both in isolation and concurrently. NoSQL database systems have recently attracted much attention, but few studies in the literature address their direct comparison with relational databases when applied to build the persistence layer of a standardized medical information system.

**Methods:**

One relational and two NoSQL databases (one document-based and one native XML database) of three different sizes have been created in order to evaluate and compare the response times (algorithmic complexity) of six different complexity growing queries, which have been performed on them. Similar appropriate results available in the literature have also been considered.

**Results:**

Relational and non-relational NoSQL database systems show almost *linear* algorithmic complexity query execution. However, they show very different linear slopes, the former being much steeper than the two latter. Document-based NoSQL databases perform better in concurrency than in isolation, and also better than relational databases in concurrency.

**Conclusion:**

Non-relational NoSQL databases seem to be more appropriate than standard relational SQL databases when database size is extremely high (secondary use, research applications). Document-based NoSQL databases perform in general better than native XML NoSQL databases. EHR extracts visualization and edition are also document-based tasks more appropriate to NoSQL database systems. However, the appropriate database solution much depends on each particular situation and specific problem.

**Electronic supplementary material:**

The online version of this article (doi:10.1186/s12911-017-0515-4) contains supplementary material, which is available to authorized users.

## Background

Electronic Health Record (EHR) Knowledge Management Systems (KMS) or EHR systems (for short) form an essential part of medical care today. However, creating, maintaining and communicating EHR documents in those systems is not at all straightforward. This is due to several factors affecting technical, syntactic and semantic interoperability between information systems, including the inevitable rapid change and evolution of medical knowledge. In order to achieve such goals, EHR systems and documents have been normalized in several international standards such as ISO/EN 13606, openEHR and HL7 [1–4].

The ISO/EN 13606 and openEHR standards define a dual model that separates information and knowledge into two levels of abstraction, thereby guaranteeing semantic interoperability between systems operating EHR documents [5].

Standardized EHR documents constitute information files that need to be maintained and stored physically in those systems. The special nature of medical knowledge that requires the separation into two levels of the dual model can have a profound effect on the way information in EHR documents is structured and how it is stored logically and physically in a database management system. The dual model used by standardized EHR documents requires the organization of the information following a specific structure, and medical knowledge must also adopt the structure constrained by the archetypes, i.e. special data structures holding knowledge [1, 2, 6–8].

Standardized EHRs are a form of big data, from which patterns may be extracted using Data Mining (DM) and Machine Learning (ML) techniques to generate new knowledge [9]. Standardized EHR extracts (see next subsection) may be extracted automatically from non-standardized EHR repositories using standard technologies based on W3C (World Wide Web Consortium) XML Schemas [10].

Cross-organizational EHR communication will constitute a key component of future health care [10]. Previous literature suggests that implementing a fully functioning EHR system with participation of all healthcare organizations could lead to a USD 77.8 billion benefit for the United States [11, 12].

### Relational approach

For decades Database Management Systems (DBMS) have been dominated by the relational model paradigm [13]. This model has a well-established theoretical background which has been well studied and understood, and has long guaranteed consistency and efficiency within database systems. However, the complex structure of the information adopted by the normalized EHR documents may cause the direct application of the relational model following this structure (Object Relational Mapping, ORM) [14] to be complicated and inefficient. Several improvements within the relational model have been proposed and used. In this paper we revise Node + Path [15], developed by openEHR, and the Archetype Relational Mapping (ARM) [16].

The Object Relational Mapping (ORM) exhaustively maps the structure of a standardized EHR extract XML (eXtensible Mark-up Language) file to a relational database [13, 17]. An EHR extract is defined as a unit of communication of all or part of an EHR document and is also an instance of the ISO/EN 13606 Reference Model (RM) [18]. ORM implies the construction of many tables related through foreign keys representing the complex structure of the extract XML file and may damage performance.

### Relational improvements

openEHR [3] promotes a Node + Path persistence solution that serialises subtrees of the whole extract XML file into BLOBs (binary latge objects) in a few relational tables, taking advantage of the semantic paths of the normalized EHR extracts. This is a simple and flexible solution, but its simplicity causes complex data retrieval logic, thereby damaging complex queries [15].

Archetype Relational Mapping (ARM) [16] is another interesting relational improvement. Node + Path uses a general data storage structure that is independent of archetypes. Another approach is to generate a database model to design a persistence layer driven by archetypes. This solution builds a new relational schema based on mappings between the dual model archetypes and relational tables.

### Non-relational NoSQL approach

All previous persistence solutions have been based upon an underlying relational database system. However the relational paradigm was recently questioned by NoSQL (document-based) database systems. A NoSQL (Not Only SQL) (SQL, Structured Query Language) database provides a mechanism for storage and retrieval of data which is modelled on means other than the tabular relations used in relational databases [19, 20]. A document-based NoSQL database system stores documents in any format like XML [21] or JSON (JavaScript Object Notation) [22] as data [23]. NoSQL DBMSs do not substitute existing relational DBMSs, but may be appropriate in specific situations. Many NoSQL databases store documents as entire BLOBs. They have no schema and do not support either joins or atomicity, consistency, isolation, or durability (ACID) properties [24]. So they may be very inefficient if a subpart of a document references parts of other such documents through an indirection link, because the whole referenced document(s) must be processed sequentially [25]. However if the main task carried out by the DBMS is a document-based task, a non-relational database may be appropriate. This is because NoSQL data stores allow stored data to remain in a form that more closely approximates its true representation [24]. And also because of the special persistence policies of EHR documents (see Discussion below).

In the specific case of standardized medical information, our problem is not the lack of a schema. In fact we have a complex and over specified schema. Recent research [26] addresses the automatic discovery of a schema in document stores in NoSQL applications in order to simplify data management and to combine both fixed-schema SQL and flexible-schema NoSQL in a single data management system [27]. Conversely, the ARM relational approach discussed in Methods below attempts to directly manage standardized medical information by simplifying its complex schema.

### NoSQL current state-of-art

There are numerous whitepapers, blog entries and commentaries on the advantages of NoSQL database systems [28]. However, there has been little research on evaluating the use of NoSQL databases in the healthcare domain [29], particularly with realistic standardized healthcare data.

NoSQL databases might offer a solution to the big amount of medical information bottleneck [30, 31].

There are over 150 different NoSQL databases, grouped into the following four categories: (1) Key-value store, (2) Document store, (3) Column-family, and (4) Graph database [32]. Open source availability of NoSQL databases reduces the overall cost considerably [29]. Within one application, different classes of NoSQL databases can be used simultaneously, which is known as polyglot persistence. In general, each class of NoSQL database is designed for a specific purpose [24, 23].

Restricting our description to document-store kind MongoDB, it provides high performance data persistence to support embedded data models which reduce I/O activity, automatic horizontal scale-up, unstructured data model (which suits EHRs), high available distributed system, denormalized localized data reducing the need for joins, 1/10 cost relative to relational SQL-based systems and better performance than these systems [33]. It also improves big data analysis performance on EHR systems over SQL-based systems [34].

Other NoSQL document-store databases such as CouchDB, used on normalized data, perform better than corresponding relational systems on non-normalized (i.e. simpler) EHR data, and are promising where ACID properties are not strictly required [35].

### Objective

This research showcases several experiments which have been carried out in order to directly compare the implementation of the persistence layer of an EHR system using three different DBMS: one relational (MySQL) and two NoSQL (document-based MongoDB and native XML eXist). Three increasing size collections of 5000, 10,000 and 20,000 real standardized EHR extracts (provided by several hospitals) have been stored, retrieved and queried on each DBMS in order to calculate their response times (computational complexity as test collection size duplicates). Concurrency experiments have also been conducted in order to compare the performance of relational MySQL and NoSQL MongoDB DBMS from that perspective.

Node + Path and ARM improvements to the relational model are also considered and discussed. Their performance is illustrated by results previously published in the literature, using similar databases and queries. In this way we get a general perspective of the most important DBMS methodologies which have been used to persist normalized medical information in current state-of-the-art EHR systems.

This research covers an investigation into the appropriateness of relational and NoSQL database systems under different situations and perspectives. The emergence and utility of NoSQL databases and its relationship to relational systems has not yet been sufficiently discussed, in the context of *standardized* medical information persistence.

## Methods

In order to directly compare different EHR extracts database persistence systems we have used examples of three of the most important database system methodologies, i.e. relational (MySQL), document-based NoSQL (MongoDB) and native XML (document-based) NoSQL (eXist) .

These DBMSs have been tested against three databases formed by 5000, 10,000 and 20,000 ISO/EN 13606 standardized EHR extracts containing alerts, problems and pharmacy information for a lower number of patients (so with a certain number of extracts per patient).

These extracts have been put together using information from several Spanish hospitals (Fuenlabrada University Hospital, Barcelona Clinical Hospital and A Coruña University Hospital Complex) and primary health care centres. These centres work with us in the PITES (Plataforma de Innovación en nuevos servicios de TElemedicina y e-Salud) coordinated research project [36]. Information comes from different departments of hospitals and health care centres. It consists of heterogeneous information residing in information systems from different manufacturers and vendors. It has been normalized, homogenized and centralized using archetype-based data transformation technologies such as the LinkEHR studio tool [37, 38]. It has also been properly anonymized using a solution developed by our Unit in previous work [39].

Queries of increasing complexity were performed on these databases, against the information contained in the problems list. Response times to these queries were calculated, in order to compare the performance and the algorithmic complexity of the three DBMSs methodologies (see below).

We also provide a short description of two separately developed and related methodologies. Their figures will be shown in the results section, in order to provide a broad perspective and an insight into database persistence on standardized medical information.

### Building a relational MySQL database system to store and query normalized EHR extracts

The relational model for database persistence [13, 17] is a very well established and mature methodology, which is paradigmatic. It is based on well-known formal relational algebra and calculus, and it has guaranteed the precision and consistency of RDBMSs (Relational DBMS) for a long time. Recently, alternative methodologies (i.e. NoSQL databases) have attracted the attention of practical database system developers [20]. NoSQL approaches can be faster and more scalable when data sizes are extremely large, or when there are no internal document references that can damage speed or data consistency. However, often these suppositions will depend on each specific project. For this reason, we have implemented a relational MySQL DBMS, in order to achieve and evaluate the persistence of ISO/EN 13606 standardized EHR extracts.

**Fig. 1:**
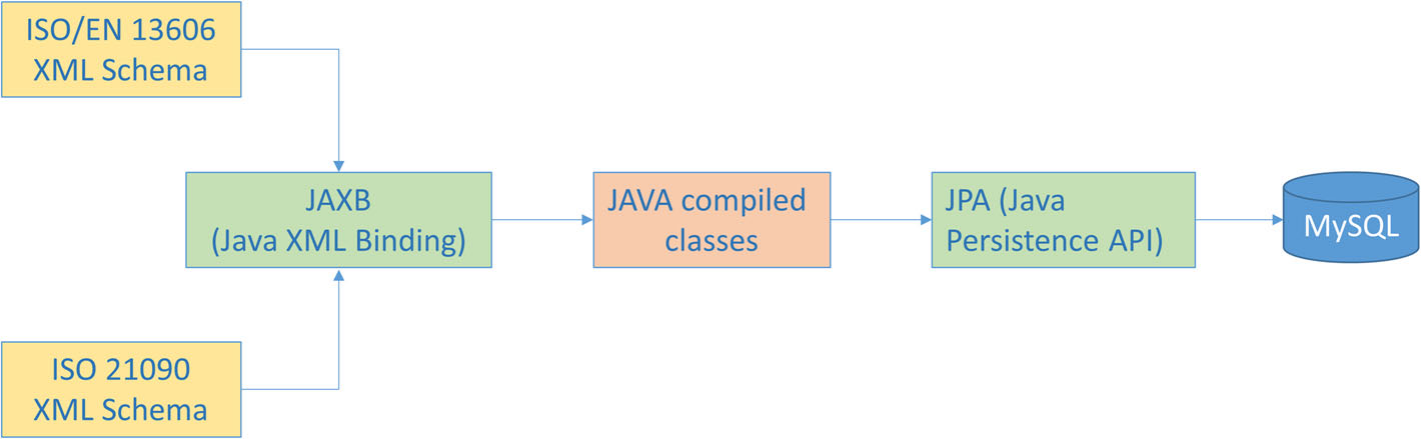
One way to perform ORM on standardized EHR extracts

We have used JAXB (Java XML Binding) and JPA (Java Persistence API) technologies in order to automatically transform the XML schemas representing the ISO/EN 13606 standard into a MySQL relational database. These XML schemas [6] indicate a standardized EHR extract system the possibly permitted XML documents this extract may adopt, i.e. its legal or valid instantiations. Consequently, JAXB can take these XML schemas as input in order to produce (as output) a Java class’s representation of any such XML extract. These Java class files may then be manually tagged with JPA codes, in order to generate a MySQL relational database with the structure of these classes, i.e. the structure of the original XML extract file. This database may be used to store, query (using standard SQL) and retrieve EHR extracts. This process is often called Object Relational Mapping (ORM) [14] and is depicted schematically in Fig. 1.

ORM may suffer the so-called “Object-Relational Impedance Mismatch”. This happens when an object is molded to fit into relational structure [23]. This motivate us to study its performance as the persistence level in an EHR system.

### Building a NoSQL MongoDB database system to store and query normalized EHR extracts

As stated above new NoSQL DBMSs (such as MongoDB [40]) have recently attracted the attention of database system developers mainly in those document-centred persistence applications, where a standard relational approach may not be efficient. As stated above this NoSQL approach may provide faster access and more especially in the case of very large databases. But it may be very inefficient if document data contain references or links to other similar pieces of documents in the database [25], and these links *affect* database consistency. In general, this NoSQL approach is adequate for a so-called document-based application.

A NoSQL database system such as MongoDB can build a database of EHR XML documents in a quite straightforward fashion. Normalized XML documents are provided as input to the system in JSON format, i.e. they must be previously transformed from XML to JSON. JSON may follow the same structure as XML. In fact the MongoDB system uses a proprietary slight variation of JSON called BSON (for Binary JSON that enables binary serialization on data), but there are programs which allow for easy conversion. Given a BSON version of the original XML extracts document collection, this is provided as input to MongoDB, and a working database is constructed in a fast and straightforward manner. BSON/JSON documents are then stored directly as BLOBs maintaining their structure, and they may be subsequently queried or retrieved.

MongoDB has its own DBMS including a complete set of CRUD (create, read, update and delete) operations. These operations are based on the tree structure of JSON/XML and rely on the tree path from root node to leaf nodes, where data may be stored. They may be translated to and from standard SQL statements, performing virtually every important feature.

Since MongoDB is a document-centred database it produces JSON/XML documents as output, which is an important aspect as it is very appropriate for document-based persistence tasks. Consequently if a CRUD operation is launched from a Java application, in order to query the extracts database, and if a table-like output is desired, the JSON output documents must be parsed and processed (as a subsequent part of the query), in order to produce such relational table-like results.

### Building a native XML NoSQL eXist database system to store and query normalized EHR extracts

EHR extracts are codified in XML format. Consequently, a native XML DBMS such as eXist [41] should be evaluated to implement the persistence layer of an EHR system. eXist is an open source management system entirely built on XML technology, also known as a native XML database. A native XML database also provides a mechanism for storage and retrieval of data, different from the tabular relations used in relational databases. Consequently, it may be considered as a NoSQL database. Being a semi-structured database, it stores data in the form of entire XML documents, so it may also be considered a document-based NoSQL database [19]. Considerations regarding the existence of links in EHR extracts stored in MongoDB in the previous section may also be applied to the eXist DBMS, considered as a kind of document-based application.

The EHR extracts are loaded by the eXist DBMS directly as XML files, maintaining their structure, and may be subsequently queried and retrieved, using the XQuery language, a W3C recommendation. The XQuery language is able to produce XML-format files output. This means that relational table-like output may be easily generated in the form of XML-formatted files, contrary to MongoDB, which produces entire JSON output that must be post-processed to yield relational-like output (see previous section).

### The openEHR node + path EHR extracts database system

We provide a short introduction to openEHR’s Node + Path even though we have not used it, and later we will show some results. openEHR has developed an optimization over the ORM relational methodology based on the EAV (entity-attribute-value) model [42]. The Node + Path persistence [15] is based on the serialisation of information objects (entire extract trees) into single blobs, requiring only one column in a relational database table. Additional indexing columns are added for attribute values in order to provide some query ability. This basic approach may be improved in two different ways: one (hybrid serialisation) serialising only lower level elements of the object trees, while storing transparently upper level objects (requiring some object-relational mapping, i.e. new tables) and another one (Node + Path approach) recording the path (as in the archetype) of each blob in a two-column table of *<node path, serialised node value >* with an index on the path column. The two improvements may be combined in a hybrid serialization Node + Path approach (see schematic representation in [15]).

This approach has the advantage of a tabular relational structure with less tables and join operations, and the direct query of fine-grained data using the paths extracted from archetypes. However, issues still remain about the uniqueness of data (archetype-based paths), fast parsing and comparison of paths, and the processing of complex queries.

### The archetype relational mapping (ARM) persistence solution

A different approach is adopted in the so-called ARM process in order to optimize the ORM relational system [16]. We will refer to it in this section since we will be presenting some results later.

In the ARM process, a new relational database schema, different from the direct relational schema used in the ORM, is generated. This means that information elements of an EHR extract as constrained and represented in the archetypes, are mapped into tables, key, foreign key and common columns. The structure of the information as stated in the archetypes is used to define the new relational schema, using these mappings.

Using this methodology, archetypes are mapped into tables, and archetype basic data types are mapped as common columns. If their occurrence in the archetype is 1, or into standalone tables with two columns, if their occurrence is higher than 1: one is a foreign key column referring identification and the other is a common column mapped form the data item. Query data items are constrained as indexed columns, in order to improve performance.

Consequently the simplified structure of the several archetypes participating in a specific EHR extract is used to build the relational schema used by that extract, instead of using the general structure of the whole RM of the dual model, as does the ORM approach.

As a result, the new simplified relational schema should be much more efficient than the straightforward (and complicated) ORM schema. This might have evident performance improvements (see the Results section).

However, since some structural information from the original extract is lost during the process of building the simplified relational schema, it is not possible to recover the original extract as it was before its storage. Thus we can query the medical information present in the extracts but we cannot recover them in their original state. This is the main reason why ARM has not been implemented in this research.

### Other relational database improvements

Other relational DBMS improvements include column-based systems [43, 44] such as for instance MonetDB [45] and VectorWise technology [46]. Column-based database systems are based on the fact that conventional row-stored systems might need to read in unnecessary data, when performing reads. On the other hand, column-stored systems only need to read in relevant data, even though writes require multiple accesses. Consequently, these systems constitute an optimization for read-intensive large data repositories. This improvement will be reasoned later in the discussion.

### Queries applied to the relational and NoSQL DBMSs

Table 1 shows six different queries that have been applied to the three size increasing databases of the three DBMSs (one relational and two NoSQL) in our experiments. Queries were performed on the extracts against the problems information of each patient. Each patient’s problem has a number of attributes such as *name*, *initial date*, *resolution date* or *severity*.

**Table 1:** Six queries performed on relational and NoSQL databases

	*Query*
Q1	Find all problems of a single patient
Q2	Find all problems of all patients
Q3	Find initial date, resolution date and severity of a single problem of a single patient
Q4	Find initial date, resolution date and severity of all problems of a single patient
Q5	Find initial date, resolution date and severity of all problems of all patients
Q6	Find all patients with problem ‘pharyngitis’, initial date > = ‘16/10/2007’, resolution date <= ‘06/05/2008’ and severity ‘high’

Queries range in complexity from *one patient* to *all patients*, from *one problem* to *all problems* for *each patient*, and from the raw list of attributes to conditions imposed on those attributes, such as dates being later than a given date or severity being ‘high’. In general terms, with the exception of Q6, the six queries are ordered by increasing complexity.

The queries were originally designed in the context of a Machine Learning application aimed at obtaining association rules held by the problems of the patients, i.e. a research application, but some were later adapted to clinical practice.

Queries Q1, Q3 and Q4 refer to problems and characteristics of problems of a single patient and are thus to be typically formulated in a scenario of clinical practice i.e. primary use of medical information.

On the other hand queries Q2, Q5 and maybe Q6 relate to problems and characteristics of problems of all the patients of the whole database and are more appropriate for secondary use of information (i.e. medical research).

Response times to queries were calculated in the relational database using MySQL 5.0.67 on Linux/SUSE. This environment yields the time in seconds of queries based on the server’s system clock. In the MongoDB database a Java application was constructed to query a MongoDB 2.6 database on Windows. The Log4j logging tool was used to set transactions at the beginning and at the end of each query to the server and total times were computed based on the timestamps of these transactions. The eXist 3.0RC1 DBMS was queried using its Java client, which yields the execution time required by each query. All values were calculated as the average response times of five query executions.

### Concurrency experiments

There are many indicators to assess performance of a DBMS. One of them is the behaviour of querying the database concurrently. In order to evaluate the ORM/MySQL and the NoSQL/MongoDB DBMSs (the NoSQL/eXist DBMS has not been included in these experiments, given the isolated queries performance results presented below) under a concurrency environment we have designed the following experiments, inspired by the XMach-1 benchmark for XML data management [47].

A Java multithread application was constructed, with three main threads representing three of the presented queries competing for CPU (Central Processing Unit) use. The queries selected were Q1, Q3 and Q4, for two main reasons: first, these are medical practice queries, which are more likely to be posed in a concurrent fashion i.e. secondary research use queries will be performed in isolation; and second, the XMach-1 benchmark recommends the use of queries with short response times (90% under 3 s) since it is rather easy to increase throughput without response time limits. For this last reason, these experiments were only performed in the small 5000 EHR extracts database.

Three different priority levels were assigned to each query, namely high, medium and low to Q1, Q3 and Q4 respectively, yielding a CPU use distribution of approximately 45%, 35% and 20%. Increasing time in milliseconds *wait* and *notify* instructions were added to decreasing priority threads in order to stabilize this distribution.

These experiments were executed five times during 10 min each. Thereafter the most executed (highest priority) query average throughput and the average response times of the three queries were calculated.

### Indexing policies

Many DBMSs build structure and range indexes automatically. For instance, in our experiments, the MySQL system built 602 such indexes in that way. We only built manually those indexes that would speed up execution of some queries. For instance, the field representing the unique identifier of a patient is a very important attribute to be indexed in queries regarding one single patient, such as Q1, Q3 and Q4. Other indexes constructed manually represent attributes demanded in our queries, such as specific problems of patients, initial and resolution dates or severity of those problems (see Table 1).

In the MongoDB and eXist systems, we also tried to construct the indexes manually. However, MongoDB emitted an error message stating that those indexes had already been constructed by the system. The eXist database did not return such an error message, but adding the indexes has not changed the response time of the queries, so we assume that those indexes were also built automatically by the system.

## Results

Table 1 shows the six different queries performed on the relational MySQL and on the two NoSQL DBMSs developed in the previous section.

Tables 2, 3 and 4 show response time in seconds of the six queries in the three DBMSs with three different database sizes, i.e. 5000, 10,000 and 20,000 normalized EHR extracts.

**Table 2:** Response times in seconds of the six queries performed on MySQL relational ORM database

*ORM*	*5000*	*10,000*	*20,000*	slope (*10 ^*−6*^ *)*
Q1	0.0429	209.0284	1082.7872	72,182.95
Q2	101.6196	>10,000	>10,000	>>
Q3	0.1256	4.1982	12.6110	832.36
Q4	0.1843	400.6388	1598.7410	106,570.45
Q5	200.8954	>10,000	>10,000	>>
Q6	0.7362	65.1898	185.2420	12,300.39
Database size	4.8GB	9.7GB	19.8GB	
Total extracts	5000	10,000	20,000	

*stands for the multiplication sign

**Table 3:** Response times in seconds of the six queries performed on MongoDB NoSQL database

*MongoDB*	*5000*	*10,000*	*20,000*	slope (*10 ^*−6*^ *)*
Q1	0.0460	0.0570	0.1221	5.07
Q2	34.5181	68.6945	136.2329	6780.99
Q3	0.0480	0.0580	0.1201	4.81
Q4	0.0520	0.0610	0.1241	4.81
Q5	38.0202	75.4376	149.9330	7460.85
Q6	9.5153	18.5566	36.7805	1817.68
Database size	1.95GB	3.95GB	7.95GB	
Total extracts	5000	10,000	20,000	

*stands for the multplication sign

**Table 4:** Response times in seconds of the six queries performed on eXist NoSQL database

*eXist*	*5000*	*10,000*	*20,000*	slope (*10 ^*−6*^ *)*
Q1	0.6608	3.7834	7.3022	442.76
Q2	60.7761	129.3645	287.362	15,105.73
Q3	0.6976	1.7710	4.1172	227.96
Q4	0.6445	3.7604	7.3216	445.17
Q5	145.3373	291.2502	597.7216	30,158.93
Q6	68.3798	138.9987	475.2663	27,125.82
Database size	1.25GB	2.54GB	5.12GB	
Total extracts	5000	10,000	20,000	

*stands for the multiplication sign

**Fig. 2:**
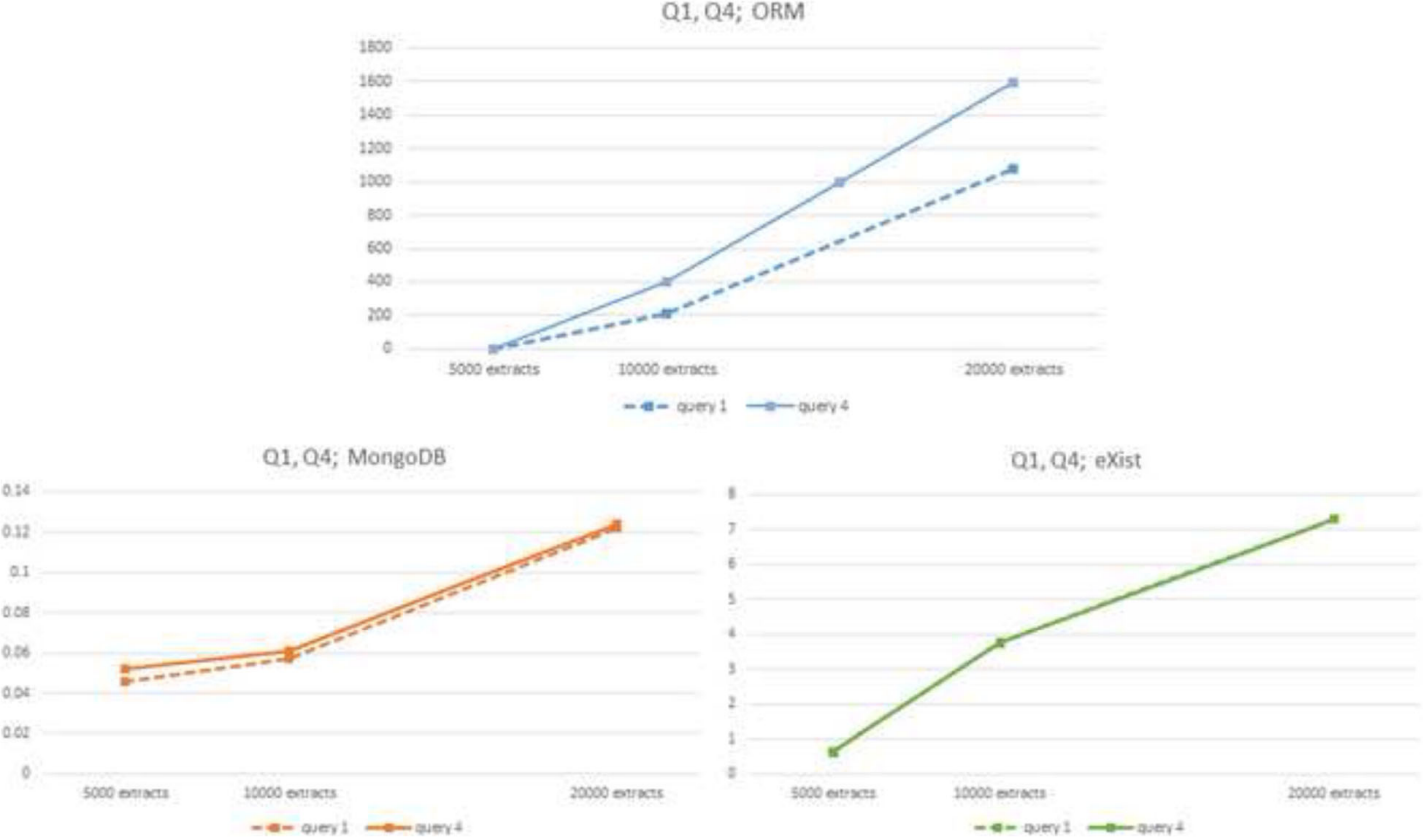
ORM (*up*) and NoSQL (MongoDB *left*, eXist *right*) response times to queries Q1 and Q4 for three database sizes

**Fig. 3:**
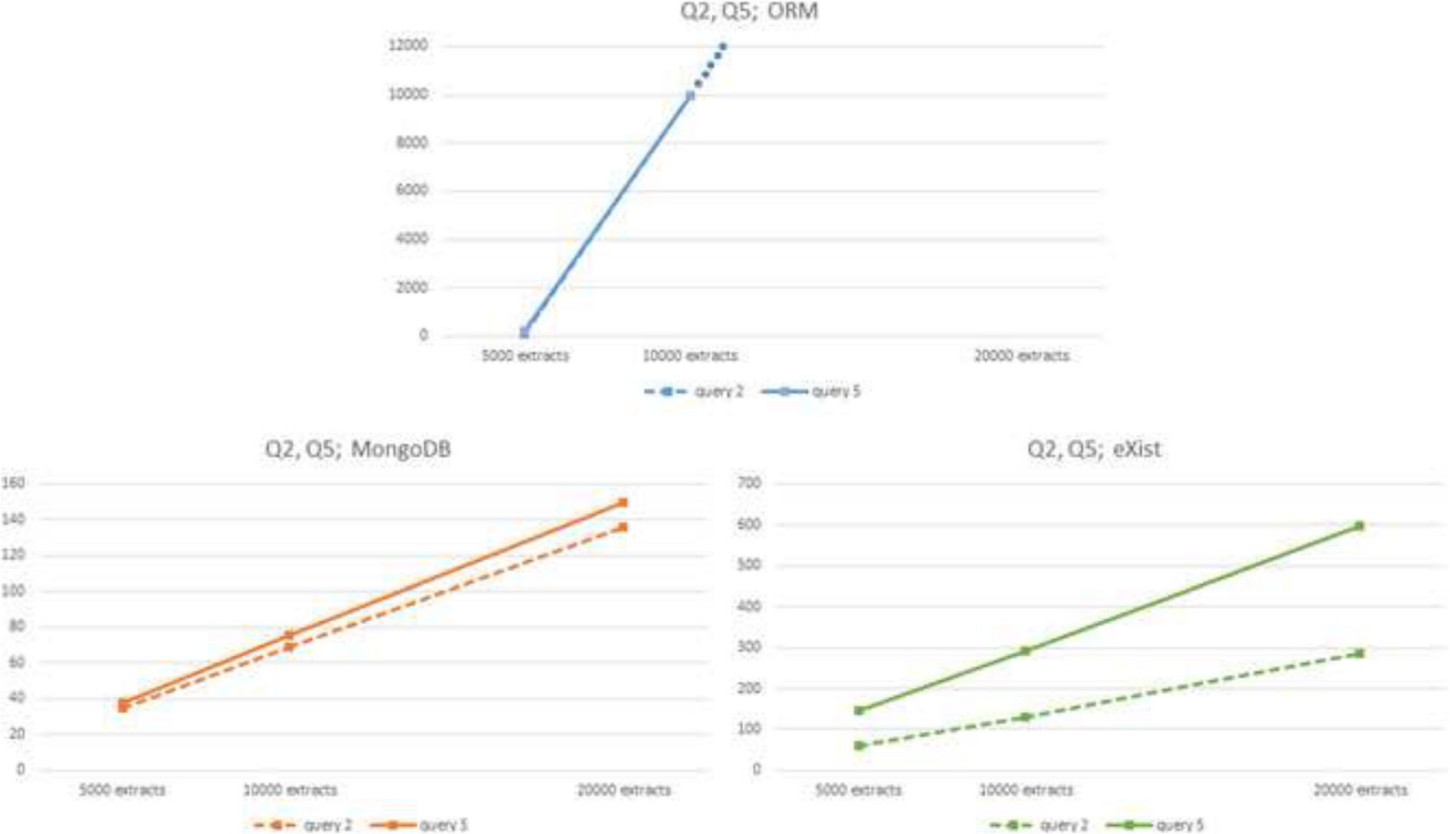
ORM (*up*) and NoSQL (MongoDB *left*, eXist *right*) response times to queries Q2 and Q5 for three database sizes

**Fig. 4:**
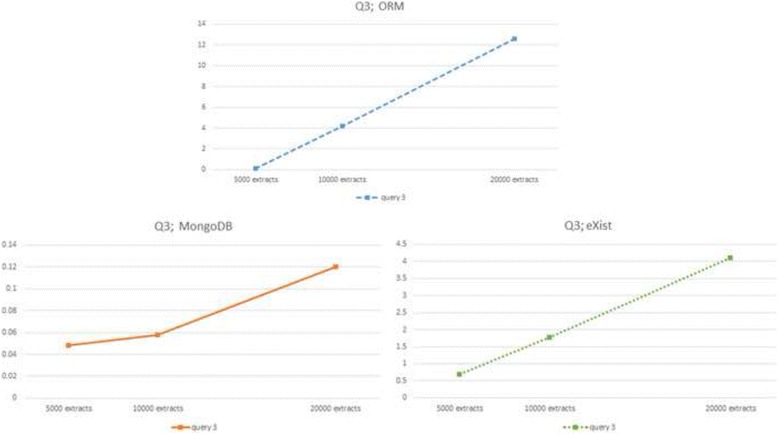
ORM (*up*) and NoSQL (MongoDB *left*, eXist *right*) response times to query Q3 for three database sizes

**Fig. 5:**
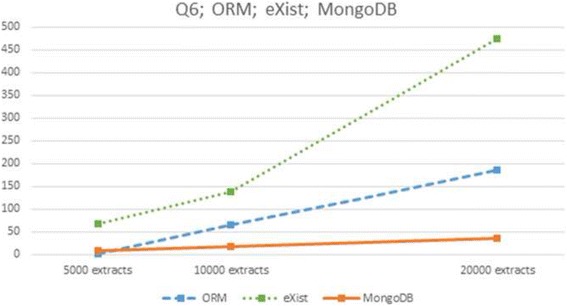
ORM and NoSQL (eXist and MongoDB) response times to query Q6 for three database sizes

At first glance one can see a strong *linear* increment in response times of the three DBMSs as the size of the database grows. This linear behaviour may be better appreciated in the nine diagrams of Figs. 2 through 5 (notice the different vertical axes scales used throughout most of these figures). Figure 2 shows queries Q1 and Q4 almost linear complexity in ORM (up), MongoDB (down left) and linear complexity in eXist (down right). Figure 3 presents linear complexity in MongoDB and eXist for queries Q2 and Q5 and unbounded response time in ORM for these queries. Figure 4 shows almost linear complexity for Q3 in MongoDB and linear complexity in ORM and eXist. Finally Fig. 5 displays linear complexity for Q6 in both ORM and MongoDB, and almost linear complexity in eXist.

However, the three DBMSs show very dissimilar slopes in their linear behaviour (see slope column in Tables 2, 3 and 4). Whilst MySQL and MongoDB yield very similar results in the small 5000 extracts database they diverge considerably in the big 20,000 extracts database, the relational being much slower than the non-relational. eXist presents slower response times than MySQL and MongoDB in the small 5000 extracts database, but intermediate slopes, thus beating MySQL in the big 20,000 extracts database, with the exception of Q6, in which ORM/MySQL behaves better.

Table 5 complements the response times of the three DBMSs showing the time and space costs of storing and retrieving EHR extract XML files on them. These storage times include the indexes being constructed or updated. The contrast between the fast time costs to store and retrieve documents in the NoSQL databases and the low response times in the relational database, and the comparable memory space costs of the NoSQL systems and the relational database is evident. This table also shows the average size in memory of one extract in the database and the average size of one extract in XML format.

**Table 5:** Shows space and time costs to store and retrieve XML documents in the three DBMSs

	*Retrieval time (ms)*	*Storage time (ms)*	*Size in memory (KB)*	*XML File size (KB)*
*ORM/MySQL*	6188.5	7569.7	960	244.876
*NoSQL/MongoDB*	14.0	35.0	390	244.876
*NoSQL/eXist*	4.9	90.9	250	244.876

### Results by other improved relational systems

We provide in Table 6, for illustrative purposes, the results obtained by the two improved relational systems (ARM and Node + Path) described in Methods above, as they appear in [16]. Our Table 6 shows the most similar queries from Table 5 in that work corresponding to three of our queries from our Table 1, with their corresponding response times.

**Table 6:** Shows illustrative data from three relational database management systems presented in [16]

	*ARM paper*	*IV(sec)*	*ARM(sec)*	*Node + Path(sec)*
Q1	Query 2.1	0.221	0.191	24.866
Q3	Query 3.1	0.242	0.270	294.774
Q6	Query 7.1	14.582	1.293	41.217
	Database size	1.60 GB	2.90 GB	43.87 GB
	Total extracts	29,743	29,743	29,743

Direct comparison with our results is not possible since database sizes are different as is the total number of extracts. This means that, for instance, since two comparable normalized relational systems (optimized ARM and ORM, 5000 extracts database) have quite similar database sizes (2.90 GB and 4.8 GB respectively), but the former holds a much larger number of extracts (29,743), the size of the extracts used in our developments (244.876 KB in XML format, see Table 5) should be much larger than that of the normalized EHR extracts used in the experiments reported in [16].

However, if we compare optimized relational system ARM (Table 6) and non-relational MongoDB system (Table 3) we can see that the latter beats the former in both Q1 (Query 2.1) and Q3 (Query 3.1): interpolating MongoDB Q1 and Q3 from the 10,000 and 20,000 extracts results to a hypothetic 30,000 extracts database (similar to the 29,743 extracts ARM database) response times would be 0.1872 and 0.1822 respectively (against 0.1910 and 0.2700 for ARM), even though ARM database size is quite a bit smaller than the 20,000 extracts MongoDB database, but also than the 20,000 extracts relational ORM database, i.e. the size of the ARM extracts should be smaller. However, comparing Q6 with query 7.1, optimized ARM performs better than the interpolated MongoDB database: interpolating MongoDB Q6 to 30,000 extracts would yield response time 55.0044. Notice from Tables 2 and 3 that Q6 is also the query where non-optimized relational ORM scores the best slope relation with respect to NoSQL.

### Results of the concurrency experiments

Tables 7 and 8 show the average throughput of Q1 (the most frequent query) and the average response times of Q1, Q3 and Q4 yielded by the concurrency experiments described in Section 2.8. Q1 achieves much higher throughput in the MongoDB setting than in the relational database. It should be noted that all three queries yield much faster response times in MongoDB than in MySQL. It seems that concurrent execution favours MongoDB, since these queries execute faster concurrently than in isolation.

**Table 7:** Shows most frequent query throughput and response times in concurrent execution in MySQL

*ORM*	*Throughput*	*Response time*
Q1	4711.6	0.0793
Q3	4711.6	0.1558
Q4	4711.6	0.9674

**Table 8:** Shows most frequent query throughput and response times in concurrent execution in MongoDB

*MongoDB*	*Throughput*	*Response time*
Q1	178,672.6	0.0030
Q3	178,672.6	0.0026
Q4	178,672.6	0.0034

## Discussion

### Direct comparison of results

We observe from the results shown in Tables 2, 3 and 4 that the relational and NoSQL database systems use very different storage and access philosophies. The very high number of tables generated in the pure relational ORM approach induces many expensive join operations, resulting in a higher computational cost as the size of the database grows and showing a much higher linear slope. In contrast, NoSQL time costs also seem to grow linearly with database size, even though with a much flatter slope. With the results obtained in Tables 2, 3 and 4 pure relational ORM does not seem practical since response times grow (almost) *linearly* but at a prohibitive slope, and will likely need improvements. On the other hand the much flatter linearity of the NoSQL systems merits further research, in order to decide the appropriateness of document-based database approaches.

Direct comparison of Tables 3 and 4 show that MongoDB performs considerably better than eXist in the six queries, yielding a linear but considerably flatter slope in all cases, and suggesting that document-based NoSQL databases such as MongoDB are a better solution than native XML NoSQL DBMSs such as eXist in order to persist and query ISO/EN 13606 standardized EHR extracts.

From Tables 7 and 8 we can see that a NoSQL MongoDB database yields much higher throughput than ORM MySQL and also query execution time is also much faster in the former than in the latter, for the six types of queries.

In fact, MongoDB queries run faster in concurrency than in isolation. This is because MongoDB query execution contains a considerable amount of time consuming administrative and communication tasks that are optimized in an execute-once fashion in the concurrent version, concentrating CPU execution time in the query itself. From this point of view, MongoDB stands as a very efficient, optimizable and effective database system.

### Table-like vs. document-like results

Results in relational databases are always presented in a table-like form, i.e. an SQL-like query always returns a set of values in the form of a relational table or similar. A whole document may also be reconstructed, but this is a fairly slow task, at least in ORM. On the other hand, a query in a document-based DBMS such as MongoDB might return a whole document (or a modified or simplified document) as result (usually XML/JSON documents); but this document may also be further processed, in order to produce a relational table-like result.

However, when medical practitioners make primary use of medical information (clinical practice), they tend to *visualize* normalized medical information regarding a *single patient*. This favours use of the queries regarding a single patient (Q1, Q3, Q4) which are about a thousand times faster than the rest of the queries in NoSQL, and the documents returned are ready for visualization. Usually, document-based NoSQL queries perform operations (projections) directly on the original whole document, using XPath-like paths that favour document generation and visualization.

A MongoDB query might thus be considered as another form or as a first step in document visualization. This visualization might interact with under-development normalized information visualization mark-up languages [48] [49]. By the same token this *visualization query* may be posed using a GUI presented to the user, or would be added as another feature of the mark-up visualization language.

### Relational vs. NoSQL database systems

We distinguish in this subsection between clinical practice i.e. primary use of medical information, and research oriented practice i.e. secondary use of it.

Regarding primary use, a probable clinical practice scenario is that formed of several extracts from a single patient, with which the medical professional is working at any given time.

In this scenario, a quite small number of EHR extract documents might be easily recovered from the database. This is a clear example of a query returning whole extracts documents, i.e. not relational tables (the kind of query best managed by a NoSQL system). In Table 1 the most similar queries to this scenario would be Q1, Q4 and maybe Q3 (see *queries applied* in Methods above), which perform better in the NoSQL databases (Tables 2 and 3). The whole extracts documents or their subsets returned by the NoSQL system are to be retrieved and visualized by the medical professional.

These documents will probably have links pointing to subparts of other such documents. These links may indicate causality or other (time) relationships between medical episodes of the same patient, and the medical professional may visualize their content navigating through them using appropriate languages, and distinguishing between their persistent or their event data [50].

When there exist links between documents, an update of a referenced element will require a join operation in a relational system, something that NoSQL databases are unable to do, compromising efficiency and consistency [25]. However, this might be a clear example of an application in which the existence of links between different documents and their subparts does *not* affect the core functionality and consistency of the application (see *building a MongoDB database* in Methods above). This is mainly because, if there is an update of some of these data or elements during such medical attention, a *new extract* should be generated with new information (data elements) and their appropriate links, without overwriting any previous data elements. This is a strict requirement of medical information: clinical data may not be overwritten, because somebody may have taken medical decisions based on it. If we are to build a link between some existing element and the new generated data element, this conforms to the usual behavior of documents visualization and edition, during clinical practice.

The information pertaining to a single patient is most easily isolated from the rest of the information in the database using a document-based rather than a relational system. However, it might be argued that sometimes a query requires the whole database. For instance, a medical professional might pose a query such as ‘show me the diagnoses given to patients with symptoms A, B and C’. This query requires the entire patients’ database, not just the documents pertaining to a single patient. This situation, which should be common enough, is usually dealt with by treating it as *segregated knowledge*, which is pre-packaged for the medical practitioner’s use, avoiding whole database time-consuming queries.

In relation to secondary or research use of medical databases, the existence of links between parts of the EHR XML documents should be transparent to the underlying database technology, be it relational or document-based. More or less complicated ‘epidemiological’ queries may be performed on a NoSQL or on a relational database, often obviating these links. In the case of MongoDB implementing an extremely big database, its apparent flatter *linear* behaviour would favour it versus a relational approach, in which joins of ever growing relational tables would produce high-slope linear complexity.

From the results of this research, it is evident that direct ORM should be improved. ARM improves ORM in two ways: (a) it diminishes relational table size by using archetypes that subdivide such tables into subparts, each corresponding to a different archetype (this is very important because relational systems perform expensive joins whose complexity grows very rapidly with table size) and (b) by designing a new relational model it is also able to diminish the number of different tables that represent extracts knowledge. However, assume that we are able to diminish table size by 10 times, using 10 different archetypes, then as soon as our database is big enough again (10 times bigger) we will be back in the situation of ORM. In other words, the number of different archetypes does not grow as fast as database size. Regarding (b), it is not clear in [16] how the original structure of the extract should be recovered, since the new relational model has strongly changed and simplified it.

This hypothesis is confirmed in [51]. The NoSQL systems evaluated in [51] show simpler linear complexity (as does MongoDB) while the MySQL relational system time responses grow much faster, even though this particular system holds non-normalized, simpler data. This fact confirms the fundamental results suggested by Tables 2 and 3, and will have severe consequences as the size of the database gets bigger. Notice also that the sizes of the databases presented in that work are not extraordinarily big (in fact the relational MySQL database is quite small; the largest NoSQL databases are not as big as expected, in order to hold 600,000 records/extracts), i.e. if a next-step bigger database were tested some NoSQL systems would probably perform better than the simple non-normalized relational MySQL system presented (see Fig. 1 and Table 1 in [51]).

The fact that query Q6 in Table 3 (NoSQL) is outperformed by optimized ARM (query 7.1 in Table 5), see *results by other improved relational systems* in Results above, is consistent with the result derived from Tables 2 and 3. Q6 is the query with by far the lowest relational/non-relational slope ratio and is thus also consistent with the hypothesis (confirmed by Q1 and Q3 in section 3.1) that relational systems are *in general* algorithmically more complex than non-relational systems and that for very big databases NoSQL outperforms (optimized) relational systems.

However, one limitation of this study is the availability of direct results for queries similar to Q2 and Q5 (secondary or research use; and considering Q6 as a non-pure secondary-use query, i.e. in the middle between primary and secondary use) applied to the ARM system. [16] does not provide such results. We rely at the moment on the results provided by Tables 2 and 3 and [51], as well as the results in [16] discussed above, to maintain our hypothesis until such experiments are performed.

While ARM improvements are considerably ‘algorithmic’, the optimization presented in column-based relational databases such as VectorWise is more hardware-oriented. Consequently, even though column-based systems need not simplify the relational model as does ARM, they are still more vulnerable to database size growing.

The linearity of NoSQL (MongoDB) and relational (in this case object-relational PostgreSQL) performance as database size grows is confirmed in other results in the literature such as those shown in Fig. 5 in [24]. This figure also shows clearly the considerably steeper linear slope of the relational approach relative to its NoSQL counterpart.

The different scalability of the DBMSs is another factor playing an important role: relational systems scale vertically (scaling-up) i.e. if the research database grows, the whole relational model must reside in the same machine. NoSQL systems scale horizontally (scaling-out), however, i.e. as the database grows it may be distributed among several machines [23]. This opens up the possibility for several CPUs to work simultaneously, thereby speeding up the execution time of ‘epidemiological’ queries several-fold.

## Conclusions

This research work has three main conclusions:

- (Non-optimized) relational model-based databases and NoSQL document-based databases both behave (almost) linearly as database size grows. However one of the former presents a much steeper slope than two of the latter. This fact has important consequences regarding database size: if it is not very big, (improved) relational databases perform reasonably well, but if it is extremely high i.e. for instance in ‘epidemiological’ queries on secondary use (research), NoSQL databases will in many cases constitute a better solution. By the same token, document-based NoSQL solutions such as MongoDB perform considerably better than (document-based) native XML NoSQL databases such as eXist.
- Standardized medical information visualization and edition is a document-based task, performed in a very small subset of the whole database. To this end, NoSQL systems fit better for several reasons, including information manageability and intuitive processing, but also database consistency is not compromised.
- Document-based NoSQL systems such as MongoDB surpass relational systems such as MySQL under a concurrent execution regime, both in throughput and in query execution time. In addition, MongoDB behaves considerably better in concurrency than in isolation. It optimizes query execution in concurrency and stands as an impressive database system from this perspective.

A fourth global corollary may also be proposed, i.e. that there is not a ‘better’ persistence solution. It depends strongly on the specific situation and problem to be solved. For instance we could implement an efficient relational system in a not very big database using optimized ARM, but then reject it and instead use a NoSQL approach, if we needed to recover the EHR extracts in their exact original form, i.e. not just query their medical information. There are many different persistence situations and scenarios and an appropriate solution should be adopted for each particular case. There are several pros and cons, but in many cases a trade-off solution is best.

## Additional files

Additional file 1: SQL program. Program written in SQL performing the six queries on the MySQL database. (SQL 15.3 kb)

Additional file 2: Java program. Program written in Java performing the six queries on the MongoDB database, using the MongoDB query language, and further processing the JSON result in order to produce relational table-like results. (JAVA 32.6 kb)

Additional file 3: XQuery program. Program written in XQuery performing the six queries on the eXist database. (XQUERY 5.51 kb)

## Abbreviations

ACID: Atomicity Consistency Isolation Durability
ARM: Archetype Relational Mapping
BLOB: Binary Large Object
BSON: Binary JSON
CPU: Central Processing Unit
DBMS: Database Management System
DM: Data Mining
EAV: Entity Attribute Value
EHR: Electronic Health Record
JAXB: JAva XML Binding
JPA: Java Persistence API
JSON: JavaScript Object Notation
KMS: Knowledge Management System
ML: Machine Learning
NoSQL: Not Only SQL
ORM: Object Relational Mapping
PITES: Plataforma de Innovación en nuevos servicios de TElemedicina y e-Salud
RDBMS: Relational DBMS
RM: Reference Model
SQL: Structured Query Language
W3C: World Wide Web Consortium
XML: eXtensible Mark-up Language
